# Selective Dual Inhibition of TNKS1 and CDK8 by TCS9725 Attenuates STAT1/β-Catenin/TGFβ1 Signaling in Renal Cancer

**DOI:** 10.3390/cimb47060463

**Published:** 2025-06-17

**Authors:** Majed Saad Al Fayi, Mishari Alshyarba

**Affiliations:** 1 Department of Clinical Laboratory Sciences, College of Applied Medical Sciences, King Khalid University, Abha 31982, Saudi Arabia; 2 Department of Surgery, College of Medicine, King Khalid University, Abha 31982, Saudi Arabia; drmhalshyarba@gmail.com

**Keywords:** renal cancer, CDK8, TNKS1, dual inhibition, WNT/β-catenin

## Abstract

Background: Tankyrase (TNKS1) regulates the WNT/β-catenin pathway, while CDK8 is a transcriptional regulator overexpressed in renal cell carcinoma (RCC). This study aims to identify novel dual inhibitors of tankyrase and Cyclin-dependent kinase 8 (CDK8), utilizing bioinformatics and in vitro methods and to assess their efficiency in renal cancer cells. Methods: To identify leads, the ChemBridge library was screening using high-throughput virtual screening (HTVS), which was followed by protein–ligand interaction analysis, Molecular Dynamics (MD) simulation, and Gibbs binding free energy estimation. A-498, Caki-1, and HK-2 cells were employed to validate in vitro efficacy. Results: TCS9725 was discovered by HTVS with binding affinities of −8.1 kcal/mol and −8.2 kcal/mol for TNKS1 and CDK8, respectively. TCS9725 had robust binding interactions with root mean square deviation values of 0.00 nm. The ΔG binding estimate was −27.45 for TNKS1 and −27.88 for CDK8, respectively. ADME predictions favored specific small-molecule inhibition profiles. TCS9725 reduced TNKS1 and CDK8 activities with IC50s of 243 nM and 403.6 nM, respectively. The compound efficiently inhibited the growth of A-498 and Caki-1 cells with GI50 values of 385.9 nM and 243.6 nM, respectively, with high selectivity compared to the non-cancerous kidney cells. TCS9725 decreased STAT1 and β-catenin positivity in A-498 and Caki-1 cells. The compound induced apoptosis and reduced TGFβ-stimulated trans-endothelial migration and p-smad2/3 signaling in both RCC cells. Conclusions: This work provides valuable insights into the therapeutic potential of TCS9725, a dual inhibitor of TNKS1 and CDK8. Further developments of this molecule could lead to new and effective treatments for this devastating disease.

## 1. Introduction

Renal cell carcinoma (RCC) is a leading cause of cancer-related mortality worldwide, with its aggressive nature and tendency to metastasize making it particularly challenging to treat [1]. RCC cells are driven by various signaling pathways that promote tumor cell proliferation, survival, and migration. Hence, overcoming resistance to conventional therapies remains a significant hurdle [2]. Among the most critical signaling cascades in RCC are the Wnt/β-catenin pathway and the CDK8-mediated transcriptional regulation pathway, both of which play pivotal roles in driving renal cancer cell proliferation and metastasis [3]. Targeting these pathways has the potential to halt tumor growth and overcome therapeutic resistance, but selective and effective inhibitors remain under development [4]. Recent advances in the treatment of metastatic renal cell carcinoma (RCC) have shifted the clinical approach toward more personalized strategies. A recent study on RCC highlights the evolving role of surgery in this context, noting that systemic therapy is often the preferred first-line option for patients who respond favorably, potentially sparing them from surgical risks. However, for patients with operable disease that is resistant to pharmacological interventions, surgical procedures such as cytoreductive nephrectomy and mastectomy continue to play an important role in disease management [5].

Wnt/β-catenin signaling is frequently deregulated in RCC, contributing to increased cell proliferation and enhanced metastatic potential [3]. Tankyrase 1 (TNKS1) is a key regulator of this pathway, promoting the stabilization of β-catenin, which translocates to the nucleus and activates transcription factors that drive tumor growth [6]. TNKS1 inhibitors, such as XAV939 and G007-LK, have shown promise in preclinical models, but their clinical development has been limited by concerns related to off-target effects and toxicity [7]. While these inhibitors can block β-catenin stabilization and attenuate tumor progression, the challenge remains in optimizing their selectivity and minimizing potential side effects [8]. Furthermore, the inhibition of a single pathway like Wnt/β-catenin may not be sufficient to fully halt RCC progression, as tumors can often develop resistance through compensatory mechanisms involving other pathways [9].

Similarly, CDK8 plays a crucial role in regulating transcriptional programs that promote RCC cell survival and proliferation [10]. As part of the mediator complex, CDK8 activates key transcription factors, including those within the Wnt/β-catenin pathway, making it a critical target for disrupting multiple cancer-driving pathways simultaneously [11]. Several CDK8 inhibitors, such as SEN461 and CDK8/19 inhibitors, have been identified in preclinical studies, but they are still in the early phases of clinical evaluation [12]. Despite their potential, CDK8 inhibitors face limitations related to specificity, toxicity, and incomplete understanding of the full spectrum of CDK8’s role in normal cellular functions [13]. These inhibitors may also face resistance due to compensatory transcriptional regulatory mechanisms that allow tumors to bypass CDK8 inhibition and continue proliferating [14].

Given the limitations of targeting a single pathway, dual inhibition of both TNKS1 and CDK8 provides a more robust strategy to disrupt RCC cell proliferation [15]. By targeting both Wnt/β-catenin and CDK8-mediated transcriptional regulation, the inhibition of these two pathways can create a multi-pronged attack that prevents tumors from compensating through alternative signaling routes. The Wnt/β-catenin pathway, through its regulation of TNKS1, complements CDK8’s effects on transcriptional activation, and together they govern the proliferation and migration of RCC cells [16]. Inhibiting both pathways simultaneously may enhance therapeutic efficacy by preventing the cancer cells from activating alternative compensatory mechanisms, thereby reducing the likelihood of resistance [17]. This dual inhibition strategy could overcome the resistance seen with single-pathway inhibition, offering a more effective treatment approach for RCC [18].

The current study aims to explore the potential of TCS9725, a novel dual inhibitor of TNKS1 and CDK8, as a therapeutic agent against RCC. By leveraging computational approaches, including molecular docking and dynamics simulations, this study seeks to evaluate the compound’s ability to selectively target both TNKS1 and CDK8, thereby inhibiting the critical signaling pathways that drive RCC progression. The dual inhibition of TNKS1 and CDK8 holds promise for overcoming the limitations of single-pathway inhibition and may provide a more effective strategy for combating RCC proliferation and metastasis. Further preclinical and clinical evaluations will be essential to determine the efficacy and safety of TCS9725 and similar compounds in the treatment of RCC.

## 2. Materials and Methods

### 2.1. Materials

Standard CDK inhibitor Dinaciclib (#SML3722) was procured from Sigma Aldrich, Burlington, MA, USA. TCS9725 (#5107441) was purchased from ChemBridge, San Diego, CA, USA. A-498 (#HTB-44), Caki-1 (#HTB-46), HK-2 (CRL-2190), and HUVEC (PCS-100-013) were from ATCC, USA. The TNKS1 (PARP5A) Colorimetric Assay Kit (#78576), the C5aR inhibition kit (#78886), and standard compound XAV-939 (#27100) were purchased from BPS Bioscience, San Diego, CA, USA. Kinase-GloTM Max (#V6071) was from Promega Corporation, Madison, WI, USA. The QCM™ Tumor Cell Trans-Endothelial Migration Assay kit (#ECM558) was from Merk Millipore, Sheboygan Falls, WI, USA. Phospho-STAT1 (Ser727)-FITC (#MA5-37074) and the Annexin V kit were from Thermo Scientific, Waltham, MA, USA. PE Mouse anti-Smad2 (pS465/pS467)/Smad3 (pS423/pS425) (#3562586) was from BD Biosciences, San Jose, CA, USA. β-catenin monoclonal antibody–PE (#12-2567-42) was from eBioscience™, Thermo Scientific, Waltham, MA, USA.

### 2.2. Methods

#### 2.2.1. High-Throughput Virtual Screening

A high-throughput virtual screening (HTVS) technique was conducted using the SiBioLEAD platform (https://sibiolead.com/, [19]) with a ChemBridge compound library comprising approximately 850,000 molecules. The three-dimensional structure of TNKS1 (PDB ID: 2RF5) and CDK8 (3RGF) was retrieved from the Protein Data Bank (PDB) and prepared according to standard protein preparation protocols. The compound library was pre-filtered based on molecular weight (150–350 Da) and adherence to Lipinski’s Rule of Five. Molecular docking was performed using AutoDock Vina (version 1.1.2) [20], integrated within the SiBioLEAD platform, facilitating the efficient identification of potential dual target ligands.

#### 2.2.2. Molecular Dynamic Simulation

Molecular dynamics (MD) simulations were conducted to evaluate the stability and interaction dynamics of the TNKS1::TCS9725 and CDK8::TCS9725 complexes identified from the high-throughput virtual screening (HTVS) analysis. These simulations were executed using GROMACS (version 2021) [21], accessed through the SiBioLEAD MD simulation platform [22,23]. For each protein–ligand complex, the initial structures were prepared by placing the complexes in a solvated triclinic box filled with Simple Point Charge (SPC) water molecules. The system was typed with the Optimized Potentials for Liquid Simulations (OPLS) force field. To maintain ionic neutrality, sodium chloride (NaCl) was added at a physiological concentration of 0.15 M. Before the production phase, the system underwent energy minimization using the steepest descent algorithm to eliminate steric clashes and optimize the initial conformation. Following energy minimization, a two-step equilibration process was performed under isothermal–isobaric (NPT) conditions for 300 ps, ensuring system stabilization before the production phase. Full-scale MD simulations were carried out for 100 ns for both TNKS1::TCS9725 and CDK8::TCS9725 complexes, with trajectory data recorded at regular intervals for downstream analysis. The root mean square deviation (RMSD) of the protein–ligand complexes was calculated to assess their conformational stability over time. Hydrogen bond analysis was performed to evaluate the consistency and strength of interactions between TCS9725 and TNKS1/CDK8 active sites. Additionally, Gibbs free energy calculations using the molecular mechanics Poisson–Boltzmann surface area (MMPBSA) method were conducted to estimate the binding free energy of the complexes, providing insights into their binding affinities.

#### 2.2.3. ADME Predictions

The absorption, distribution, metabolism, excretion, and toxicity (ADMET) properties of the identified ligands were assessed using the ADMET-AI tool available at https://admet.ai.greenstonebio.com/ [24]. To ensure compatibility with the platform, ligands were first converted into the Simplified Molecular Input Line Entry System (SMILES) format. These SMILES representations were then uploaded to the ADMET-AI server, which performed comprehensive predictions of key pharmacokinetic and toxicity parameters. The platform provided a detailed evaluation of drug likeness, including absorption potential, metabolic stability, tissue distribution characteristics, and potential toxicity risks. Graphical visualizations of the predicted parameters facilitated the interpretation and comparison of different ligands. This analysis was essential for selecting compounds with favorable pharmacokinetic and toxicity profiles, ensuring their suitability for further preclinical investigation.

#### 2.2.4. TNKS1 Inhibition Assay

The assay was performed as per the manufacturer’s instructions, using the TNKS1 (PARP5A) Colorimetric Assay Kit (#78576) from BPS Bioscience. Accordingly, 25 µL/well of the master mix containing 2.5 µL of 10X PARP, 10 µL of PARP substrate mixture, and 12.5 μL of distilled water was added to the histone mixture pre-coated to a 96-well plate. The test wells received 5 µL of TCS9725 or XAV-939 log dilutions (ranging from 0.1 nM to 10,000 nM) at final concentrations, whereas the blank and positive control received 5 µL of diluent solution. The reaction was started by adding 20 µL of TNKS1 (1.5 ng/µL) in 1x PARP buffer to the test and positive control wells. The blank wells were treated with 20 µL of 1x PARP buffer. The plate was incubated at room temperature for one hour with gentle agitation in a dark atmosphere. Next, the plate was washed three times with 200 µL of PBST buffer in each well. Each well received 50 µL of diluted streptavidin–HRP and was incubated for 30 min at room temperature in the dark. After three washes with 200 µL of PBST solution, each well received 100 µL of the colorimetric HRP substrate. The color generation was stopped with 100 µL of 2 M sulfuric acid in each well, and the absorbance was measured at 450 nm using an Omega Fluostar microplate reader from BMG LABTECH, Cary, NC, USA. The results were analyzed with GraphPad Prism software (version 6.0), and the IC50 value was reported.

#### 2.2.5. CDK8 Inhibition Assay

Inhibition of CDK8 activity by TCS9725 was enumerated using the luminescence-based kinase assay kit as per the manufacturer’s instructions. Accordingly, 12.5 µL/well of the master mix (which contains 6 μL of 5x kinase assay buffer, 0.5 µL of 500 µM ATP, 1.25 µL of CDK Substrate Peptide, and 4.75 µL of distilled water) was added to the 96-well plate. This was followed by the addition of 2.5 µL of the log dilutions (ranging from 0.1 nM to 10,000 nM) of TCS9725 or the standard MLN-120B at the designated final concentrations to the test wells, while the blank and positive control received 2.5 µL of diluent solution. The reaction was initiated by adding 10 µL of CDK8 (15 ng/µL) prepared in 1x kinase assay buffer to the test and positive control wells. Blank wells were added with 10 µL of 1x kinase buffer. The plate was incubated at 30 °C for 45 min. An amount of 2.5 µL of Apo-Glow reagent was added to all wells. The plate was kept in the dark for 45 min at room temperature. Incubation was followed by the addition of 50 µL of kinase detection reagent to all wells, followed by another incubation for 45 min in the dark at room temperature. The plate was read for luminescence using a FLUOstar Omega microplate reader (BMG LABTECH, Cary, NC, USA). Blank values were subtracted, and the percentage inhibition of kinase activity was calculated and analyzed using GraphPad Prism software (version 6.0). Half-dose inhibitory concentration (IC50) values were presented.

#### 2.2.6. Cell Culture and Proliferation Assay

A-498, Caki-1, and Vero cells were grown in RPMI-1640 medium supplemented with 10% FBS, 100 U/mL penicillin, and 100 U/mL streptomycin. The MTT test was employed to determine proliferation, as reported before [25]. The cells were seeded in 96-well plates (5 × 10^3^ cells/well) and treated with TCS9725 log dilutions (0.1 nM to 10,000 nM) for 72 h. Following that, the cells were treated with 1 mg/mL MTT, incubated for 4 h, and then dissolved in DMSO. The absorbance was measured at 560 nm. The GI50 values were calculated by analyzing the percentage inhibition of cell growth using the GraphPad Prism 6.0 program.

#### 2.2.7. STAT1 and β-Catenin Signaling in RCC

Downstream signals of TNKS1 and CDK8 in the RCC cells with TCS9725 were analyzed using flow cytometry. We used the respective near-GI50 values of the A-498 and Caki-1 cells to assess the effect of the compound in RCC cells. A-498 cells were exposed to 386 nM, and Caki-1 cells were exposed to 244 nM, of TCS9725. Both the cell types were incubated for 12 h following the compound treatments. After the incubation period, all cells were removed from the plates and transferred to sterile Eppendorf tubes. The cells were fixed with 4% formaldehyde for 10 min and then treated with 90% methanol at −20 °C for 15 min. The cells were then incubated in 1x HBSS buffer and 10% normal goat serum to block non-specific protein–protein interactions. The cells were stained with anti-phospho-STAT1 (Ser727)-FITC antibody (1:10 dilution) or β-catenin monoclonal antibody–PE (0.25 μg/mL) for 15 min in dark. After two washes to remove the extra dye, the cells were suspended back in the HBSS buffer. A Guava EasyCyteTM flow cytometer was used to acquire 5000 events. Analysis was carried out using InCyte software (Vesrsion 0.2) from Millipore to enumerate the percentage of positive populations to compare against the untreated controls.

#### 2.2.8. Annexin V Assay

A-498 cells and Caki-1 cells were treated with 386 nM and 244 nM of TCS9725, respectively, for 48 h. Following the incubation period, the cells were washed using the kit’s buffer and stained for 15 min in the dark using 0.25 µg/mL Annexin V reagent. After two further washes, the cells were resuspended in a kit solution containing 0.5 µg/mL propidium iodide. Flow cytometry was conducted by acquiring data from 10,000 events using a Guava easyCyte system, and the results were analyzed with InCyte software to distinguish between healthy and apoptotic cells (early- and late-phase apoptosis). The findings were presented using GraphPad Prism software (version 6.0; La Jolla, CA, USA).

#### 2.2.9. Tumor Cell Trans-Endothelial Cell Migration in RCC Cells

The assay was carried out using a calorimetry-based kit of 24-well trans-well inserts (8 μm), as described previously [26]. Briefly, 1 × 10^5^ of A-498 cells and Caki-1 cells that were starved overnight in a serum-free media were transferred to the pre-grown inserts with a monolayer of the HUVEC cells. A-498 cells were exposed to 386 nM TCS9725, and the Caki-1 cells were treated with 244 nM TCS9725 and incubated for 1 h in a CO_2_ incubator. The inserts were then transferred to new wells with cell growth media containing 10 ng/mL TGF-β1. The RCC cells were now allowed to migrate across the HUVEC membrane for 6 h in a CO_2_ incubator. Followingly, the inserts were removed from the wells and stained for 15 min using the staining solution provided in the kit. Furthermore, the stain was eluted using the kit elution buffer and read for absorbance at 570 nm. Percentage inhibition of the A-498 and Caki-1 cell migration across the HUVEC membrane was calculated with reference to the control and presented.

#### 2.2.10. TGF-β-Stimulated Smad2/3 Signaling in RCC

For Smad2/3 evaluation, the A-498 and Caki-1 cells were treated with 386 nM and 244 nM of TCS9725, respectively, and incubated for 1 h in a CO_2_ incubator. Followingly, the cells were incubated with TGF-β1 (10 ng/mL) for 6 h. After the incubation period, all cells were removed from the plates and transferred to sterile Eppendorf tubes. The cells were fixed with 4% formaldehyde for 10 min and then treated with 90% methanol at −20 °C for 15 min. The cells were stained with anti-Smad2 (pS465/pS467)/Smad3 (pS423/pS425) antibody–PE (0.25 μg/mL). After two washes to remove the extra dye, the cells were suspended back in the HBSS buffer. A Guava EasyCyteTM flow cytometer was used to acquire 5000 events. Analysis was carried out using InCyte software from Millipore to enumerate the percentage of positive populations to compare against the uninduced/TGF-β-induced controls.

#### 2.2.11. Statistical Analysis

All experiments were performed in triplicate, and data were expressed as mean ± standard deviation (SD). Statistical analysis was conducted using GraphPad Prism software (version 6.0; La Jolla, CA, USA). GI_50_ and IC_50_ were calculated using a non-linear regression fit model with variable slope and plotted accordingly. Statistical difference was analyzed using one-way ANOVA followed by Tukey analysis. Statistical significance was set at *p* < 0.05.

## 3. Results

### 3.1. Structure of TNKS1

**Figure 1:**
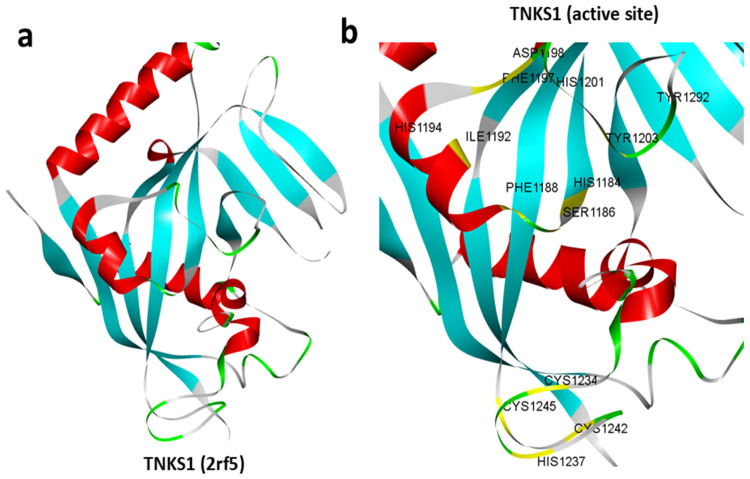
Structure of TNKS1. (**a**) Three-dimensional representation of the retrieved experimental structure of TNKS1 (2rf5). (**b**) Retrieved structure of TNKS1 showing active site residues (highlighted in yellow).

To identify the targeted small molecule against TNKS1 that has a dual interaction property with both TNKS1 and CDK8, first, the three-dimensional structure of TNKS1 was retrieved from the Protein Data Bank (PDB ID: 2rf5) and visualized to analyze its overall conformation (Figure 1a). The structure consists of multiple domains, including the ankyrin repeat domain, sterile alpha motif (SAM) domain, and poly(ADP-ribose) polymerase (PARP) catalytic domain. The molecular architecture was rendered to depict the secondary structural features, revealing a well-defined fold characteristic of TNKS1. A close-up view of the active site was generated to identify key residues involved in ligand binding and enzymatic function (Figure 1b). The active site residues were highlighted in yellow, allowing for clear visualization of the potential binding pocket. The structural representation confirmed the presence of highly conserved catalytic residues, which are critical for substrate recognition and enzymatic activity. The spatial arrangement of these residues suggests a well-defined binding cavity, which could facilitate interaction with small-molecule inhibitors; therefore, this region was targeted for high-throughput virtual screening.

### 3.2. High-Throughput Virtual Screening of the ChemBridge Library

**Figure 2:**
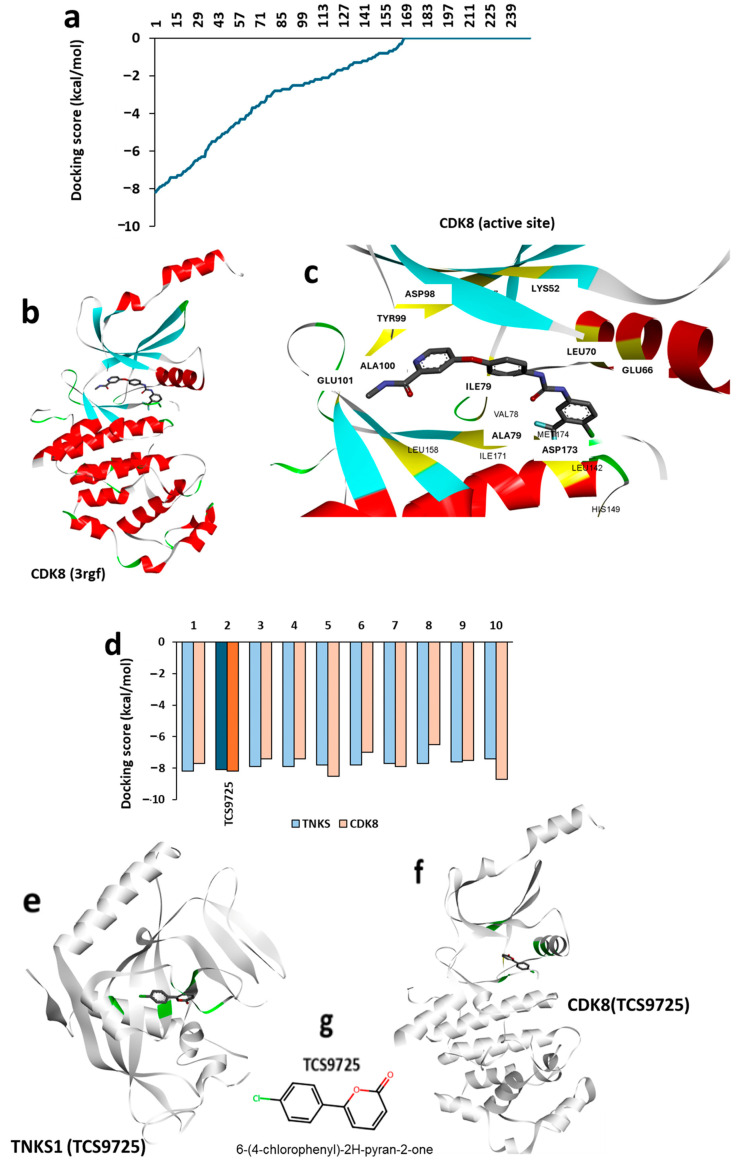
High-throughput virtual screening of the ChemBridge library. (**a**) Based on the D-HTVS method, the histogram representation shows predicted docking scores for the ChemBridge library against the TNKS1 active site. (**b**) The three-dimensional structure of CDK8 (3rgf) was retrieved from the PDB databank. (**c**) Cartoon representation highlighting the active site and its associated residues in CDK8 (yellow highlights). (**d**) Histogram showing docking scores for top 10 molecules from high-throughput virtual screening of top 50 molecules from TNKS1 screening (**a**), depicting dual interactions. (**e**) Three-dimensional representation depicting the binding of TCS9725 (top compound) with TNKS1 at its active site. (**f**) Three-dimensional representation depicting the binding of TCS9725 with CDK8 at its active site kinase domain. (**g**) Two-dimensional representation of the predicted lead molecule with dual binding property.

In order to identify top lead molecules, high-throughput virtual screening (HTVS) was performed on the ChemBridge library against the TNKS1 active site using the diversity-based high-throughput virtual screening (D-HTVS) method. The distribution of predicted docking scores is represented as a histogram (Figure 2a), showing a wide range of binding affinities across the screened compounds. To investigate potential dual-target inhibitors, the three-dimensional structure of CDK8 was retrieved from the Protein Data Bank (PDB ID: 3rgf) (Figure 2b). A cartoon representation of CDK8 was generated, highlighting the active site and its associated residues in yellow (Figure 2c). Screening results were further refined, and a histogram depicting the docking scores of the top 10 molecules from the TNKS1 screening was generated (Figure 2d), emphasizing compounds with potential dual interactions. Among the top-ranked molecules, 6-(4-chlorophenyl)-2H-pyran-2-one (internal reference number TCS9725) was identified as a promising dual inhibitor, with a reasonable binding score for both TNKS1 and CDK8. The three-dimensional representation of TCS9725 binding to TNKS1 at its active site is shown in Figure 2e, while its binding conformation at the CDK8 kinase domain is presented in Figure 2f. The 2D representation of the identified lead candidate is depicted in Figure 2g.

### 3.3. Protein–Ligand Interaction Analysis Based on Binding Characteristics of TCS9725

**Figure 3:**
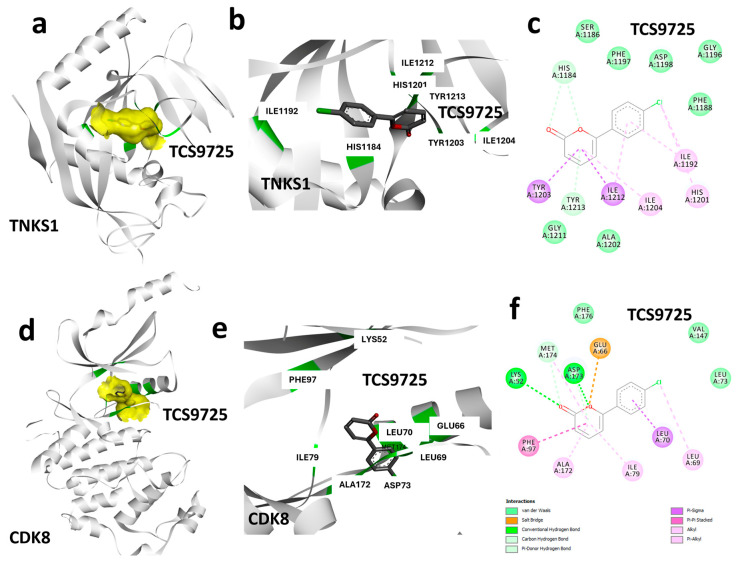
Protein–ligand interaction analysis. (**a**) Molecular surface representation depicting the ligand-binding region in TNKS1. (**b**) Cartoon representation showing the interactions of TCS9725 at the active site of TNKS1. (**c**) Two-dimensional representation showing the interactions of TCS9725 along with the nature of bonds. (**d**) Molecular surface representation depicting the ligand binding region in CDK8. (**e**) Cartoon representation showing the interactions of TCS9725 at the active site kinase domain of CDK8. (**f**) Two-dimensional representation showing the interactions of TCS9725 along with the nature of bonds.

To decipher the binding topology of the identified lead molecule, the binding interactions of TCS9725 with TNKS1 and CDK8 were analyzed using a protein–ligand interaction profiling module from Discovery Studio Visualizer. The molecular surface representation of TNKS1, highlighting the ligand-binding region, is shown in Figure 3a. A detailed cartoon representation of TCS9725 at the TNKS1 active site depicts its binding conformation and interaction network (Figure 3b). The specific interactions, including hydrogen bonds and hydrophobic contacts, are illustrated in a 2D diagram (Figure 3c). Results show, TCS9725 forms several π-alkyl and π-π interactions with TNKS1 residues, including Tyr1203, Ile1212, Ile1204, and His1201. Similarly, protein–ligand interaction profiling was performed for the CDK8-bound TCS9725 complex and results were visualized to identify the ligand-binding region (Figure 3d). The binding mode of TCS9725 at the CDK8 kinase domain is depicted in a cartoon representation (Figure 3e), showing its spatial orientation within the binding pocket. A 2D interaction diagram was generated to illustrate the key bonds and molecular interactions between TCS9725 and CDK8 (Figure 3f).

### 3.4. Molecular Dynamics Simulation Analysis of TNKS1::TCS9725 Complex

**Figure 4:**
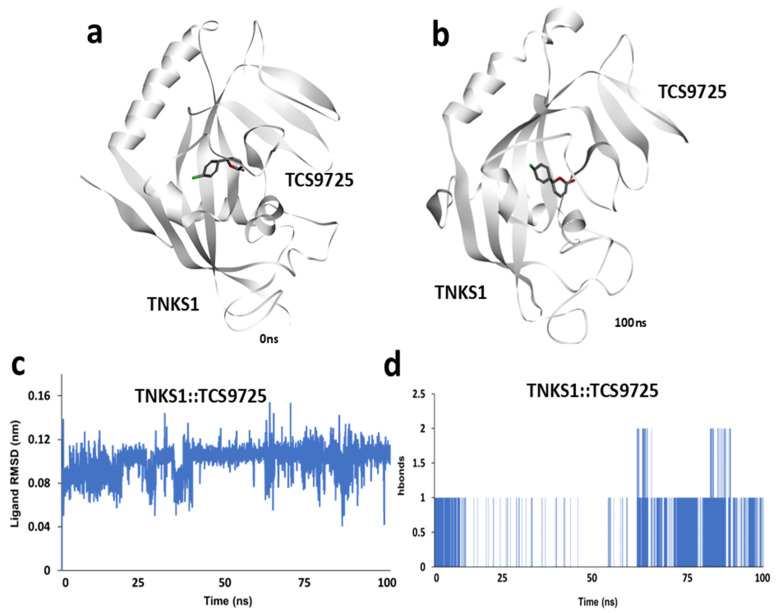
Molecular dynamic simulation of TNKS1:: TCS9725 complex. (**a**) Snapshot of simulation trajectory taken at 0 ns. (**b**) Snapshot of simulation trajectory taken at 100 ns. (**c**) Ligand RMSD calculated from 100 ns simulation trajectory and (**d**) time-course representation of average h-bonds calculated between TNKS1 and TCS9725 from 100 ns simulation trajectory.

In order to understand the stability and interaction dynamics of the TNKS1::TCS9725 complex, they were analyzed through a 100 ns molecular dynamics simulation. A snapshot of the initial conformation at 0 ns is shown in Figure 4a, depicting the initial positioning of TCS9725 within the TNKS1 binding pocket. After 100 ns of simulation, structural adjustments in both the protein and ligand were observed, as shown in Figure 4b, indicating conformational changes within the complex over time. To evaluate ligand stability within the binding site, the root means square deviation (RMSD) of TCS9725 was computed throughout the simulation. The RMSD plot (Figure 4c) displays fewer fluctuations in ligand positioning, reflecting its conformational stability and potential rearrangements within the TNKS1 active site. Additionally, the number of hydrogen bonds formed between TNKS1 and TCS9725 was monitored across the 100 ns trajectory. A time-course representation of the average hydrogen bonds (Figure 4d) provides insight into the consistency and strength of ligand–protein interactions over time. The hydrogen bond profile indicates dynamic changes in binding interactions throughout the simulation period.

### 3.5. Molecular Dynamics Simulation of the CDK8::TCS9725 Complex

**Figure 5:**
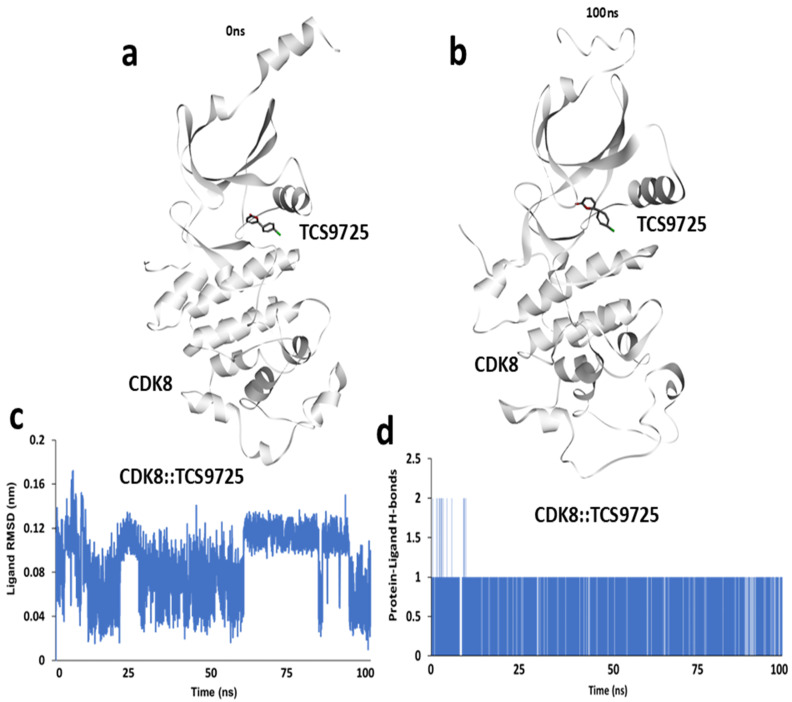
Molecular dynamic simulation of CDK8:: TCS9725 complex. (**a**) Snapshot of simulation trajectory taken at 0 ns. (**b**) Snapshot of simulation trajectory taken at 100 ns. (**c**) Ligand RMSD calculated from 100 ns simulation trajectory and (**d**) time-course representation of average h-bonds calculated between CDK8 and TCS9725 from 100 ns simulation trajectory.

The dynamic stability and interaction profile of the CDK8::TCS9725 complex were examined through a 100 ns molecular dynamics simulation. A snapshot of the initial conformation at 0 ns (Figure 5a) shows the initial positioning of TCS9725 within the CDK8 binding pocket. After 100 ns of simulation, structural adjustments in both the protein and ligand were observed, as shown in Figure 5b, highlighting potential conformational changes in the complex over time. To assess the stability of TCS9725 within the CDK8 binding site, the root mean square deviation (RMSD) of the ligand was calculated throughout the simulation trajectory. The RMSD plot (Figure 5c) illustrates fluctuations in ligand positioning, providing insights into its binding stability and dynamic behavior within the active site. Additionally, the number of hydrogen bonds formed between CDK8 and TCS9725 was analyzed across the 100 ns trajectory. A time-course representation of the average hydrogen bonds (Figure 5d) depicts variations in hydrogen bonding interactions over time, indicating the nature and persistence of key molecular interactions within the complex.

### 3.6. Gibbs Binding Free Energy Estimation and ADMET Property Predictions

**Figure 6:**
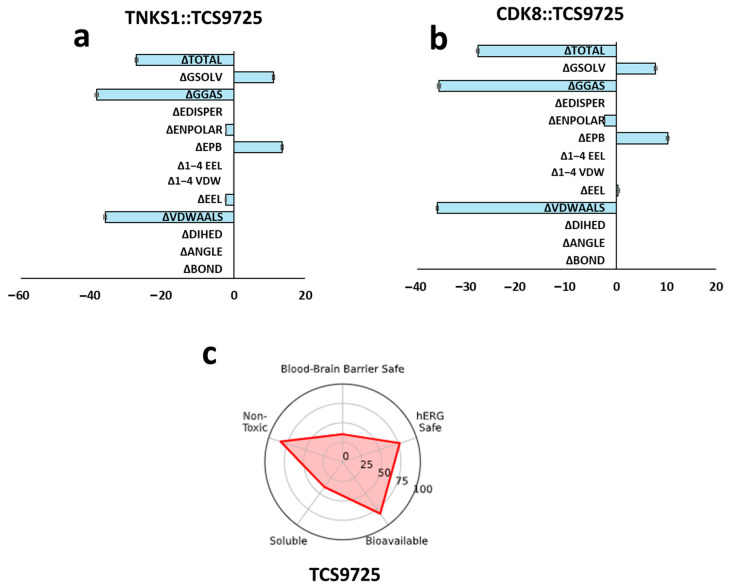
Gibbs binding free energy estimation and ADMET property predictions. (**a**) Histogram representing MMPBSA results for Gibbs binding free energy estimation from 100 ns trajectory for TNKS1::TCS9725 complex. (**b**) Histogram representing MMPBSA results for Gibbs binding free energy estimation from 100 ns trajectory for CDK8::TCS9725 complex. (**c**) Predicted ADMET properties for the lead molecule TCS9725.

The binding free energy of the TNKS1::TCS9725 and CDK8::TCS9725 complexes was estimated using the molecular mechanics Poisson–Boltzmann surface area (MMPBSA) method over a 100 ns simulation. The Gibbs free energy distribution for the TNKS1::TCS9725 complex is presented as a histogram (Figure 6a), illustrating the stability and favorability of ligand binding across the simulation trajectory. Similarly, the Gibbs free energy distribution for the CDK8::TCS9725 complex is shown in Figure 6b, providing insights into the thermodynamic stability of the ligand within the CDK8 active site. Additionally, the absorption, distribution, metabolism, excretion, and toxicity (ADMET) properties of TCS9725 were predicted to evaluate its pharmacokinetic and drug-like characteristics. The predicted ADMET properties, including parameters such as solubility, permeability, metabolism, and toxicity, are summarized in Figure 6c. These properties offer a preliminary assessment of the compound’s suitability as a potential drug candidate.

### 3.7. TCS9725 Inhibited TNKS1 and CDK8 Activities and Selectively Controlled the RCC Cell Proliferation

**Figure 7:**
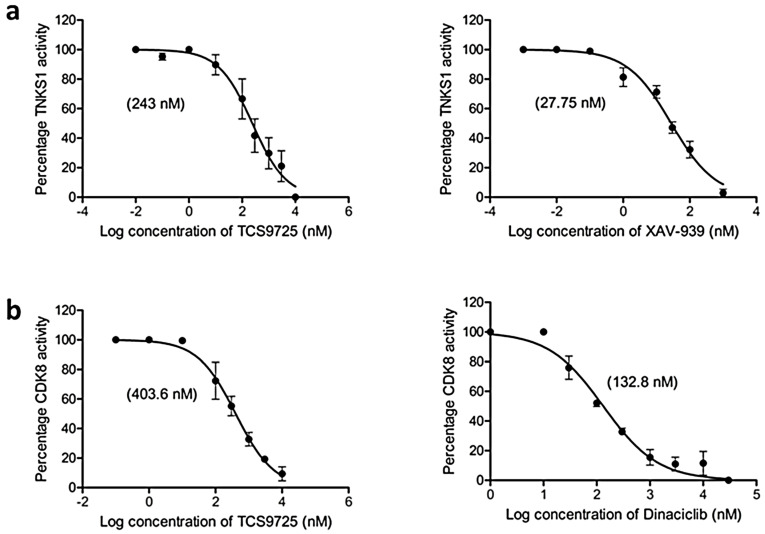
TCS9725 inhibited TNKS1 and CDK8 activity. The effect of TCS9725 and standard compounds at various concentrations was evaluated concerning (**a**) TNKS1 and (**b**) CDK8 activities and the IC50 values are presented. GraphPad Prism version 6.0 was used to examine the mean ± SD results from three studies.

To augment the in silico observations, we evaluated the inhibitory efficacy of TCS9725 against TNKS1 and CDK8 activities. The compound inhibited TNKS1 activity with an IC50 value of 243 nM (Figure 7a). The standard compound XAV-939 inhibited TNKS1 activity with an IC50 value of 27.75 nM (Figure 7a). CDK8 activity was inhibited by TCS9725 with an IC50 value of 403.6 nM, while the standard CDK8 inhibitor dinaciclib demonstrated an IC50 value of 132.8 nM against the enzyme (Figure 7b).

**Figure 8:**
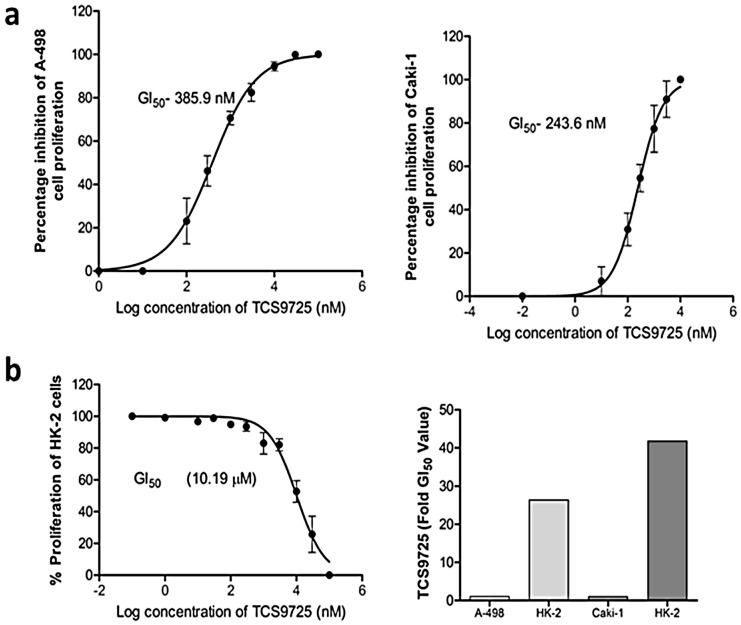
TCS9725 inhibited the RCC cell proliferations selectively. The antiproliferative activity GI50 values for TCS9725 on (**a**) A-498 cells and Caki-1 cells are shown. The compound dose-dependently inhibited the proliferation of these cell lines. (**b**) The effect of TCS9725 on the proliferation of non-cancerous, normal HK-2 kidney cells and the fold difference of the TCS9725’s GI50 value of HK-2 cells versus A-498 cells and Caki-1 cells are presented. GraphPad Prism version 6.0 software was used to examine the mean ± SD values of the percentage of cell proliferation inhibition.

We next checked the activity of TCS9725 against the proliferation of RCC cells. The antiproliferative effect of the compound was tested in A-498 cells and Caki-1 cells. TCS9725 inhibited the proliferation of A-498 cells and Caki-1 cells, with respective GI50 values of 385.9 nM and 243.6 nM (Figure 8a). The effect of the compound on normal, non-cancerous HK-2 cell proliferation was found to be inhibited at 10.19 μM (Figure 8b). The compound inhibited HK-2 cell proliferation at 26.4-fold- and 41.83-fold-higher GI50 values when compared with A-498 and Caki-1 cells, respectively (Figure 8b). We used the respective GI50 values of the compound (386 nM for A-498 cells and 244 nM for Caki-1 cells) for further assays.

### 3.8. TCS9725 Reduced the Phospho-STAT-1- and β-Catenin-Positive Population in RCC Cells

**Figure 9:**
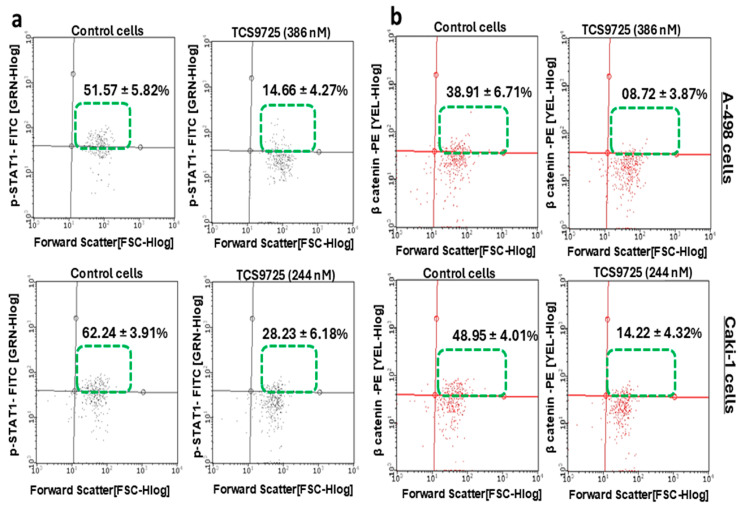
The phospho-STAT-1- and β-catenin-positive population in RCC cells was suppressed by TCS9725. The endogenous phospho-STAT-1(Ser727) population (**a**) and the β-catenin population (**b**) were reduced in A-498 and Caki-1 cells by TCS9725 when compared to the respective untreated control cells. Representative graphs are presented. Numerical values are mean ± SD from three individual experiments. Statistical significance at *p* < 0.05.

The effect of TCS9725 on the endogenous downstream signaling proteins of CDK8 and TNKS-1 was evaluated in A-498 and Caki-1 cells. The compound decreased the p-STAT-1-positive population in both RCC cells. In A-498 cells, 386 nM treatment of TCS9725 reduced the p-STAT-1-positive population to 14.66 ± 4.27% from 51.57 ± 5.82% of the untreated control (Figure 9a). Treatment with 244 nM of TCS9725 in Caki-1 cells reduced the p-STAT-1-positive population from 62.24 ± 3.91% to 28.23 ± 6.18% compared to control (Figure 9a). Similarly, in Caki-1 cells, 386 nM treatment of TCS9725 reduced the β-catenin-positive population to 08.72 ± 3.87% from 38.91 ± 6.71% of the untreated control (Figure 9b). Treatment of 244 nM of TCS9725 in Caki-1 cells reduced the β-catenin-positive population from 48.95 ± 4.01% to 14.22 ± 4.32% compared to control (Figure 9b).

### 3.9. TCS9725 Induced Apoptosis and Reduced the TGFβ-Stimulated Migration in the RCC Cells

**Figure 10:**
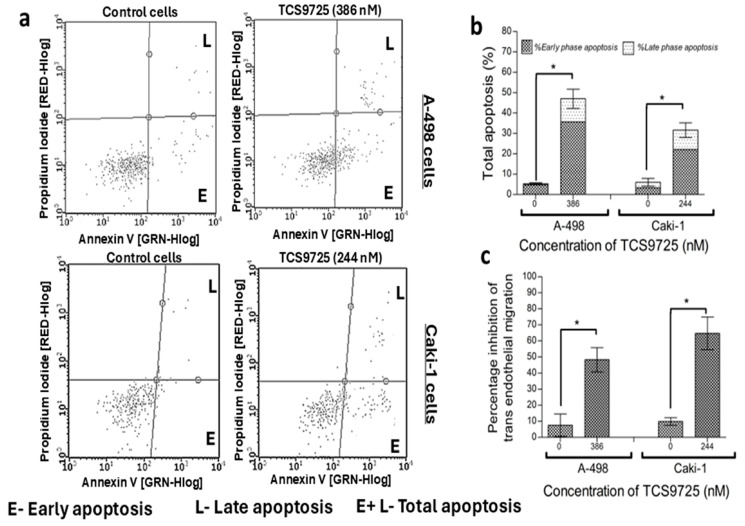
Shows the effect of TCS9725 on RCC apoptosis and trans-cell migration. TCS9725 triggered both early-phase and late-phase apoptosis in A-498 and Caki-1 cells after 48 h of treatment. (**a**) Representative Annexin V test graphs showing TCS9725 inducing early and late apoptosis in A-498 and Caki-1 cells. (**b**) Histograms showing the effectiveness of TCS9725 in inducing early, late, and total apoptosis in RCC cells. (**c**) The compound effectively inhibited the migration of A-498 and Caki-1 cells across the HUVEC membrane in the presence of 10 ng/mL TGF-β, a chemoattractant. * *p* ≤ 0.05 was considered statistically significant. Results were reported as mean ± SD from three experiments.

Treatment with TCS9725 enhanced the number of early- and late-phase apoptotic cells in the RCC cells, eventually increasing total apoptosis (Figure 10a). The compound, when treated with 386 nM and 244 nM, raised total apoptosis to 47.01 ± 8.92% and 31.60 ± 6.92% in A-498 and Caki-1 cells, respectively, while the respective controls exhibited 4.70 ± 1.11% and 6.05 ± 3.03% of total apoptotic populations (Figure 10b). The antimetastatic activity of TCS9725 in RCC cells was assessed using the TGF-β-induced trans-endothelial cell migration assay. TCS9725 substantially reduced the endothelial transmigration of A-498 cells and Caki-1 cells across the HUVEC cell membrane in response to the TGF-β stimulant (Figure 10c).

### 3.10. TCS9725 Downregulated TGF-β-Stimulated Smad2(pS465/pS467)/Smad3 (pS423/pS425) Signaling in RCC Cells

**Figure 11:**
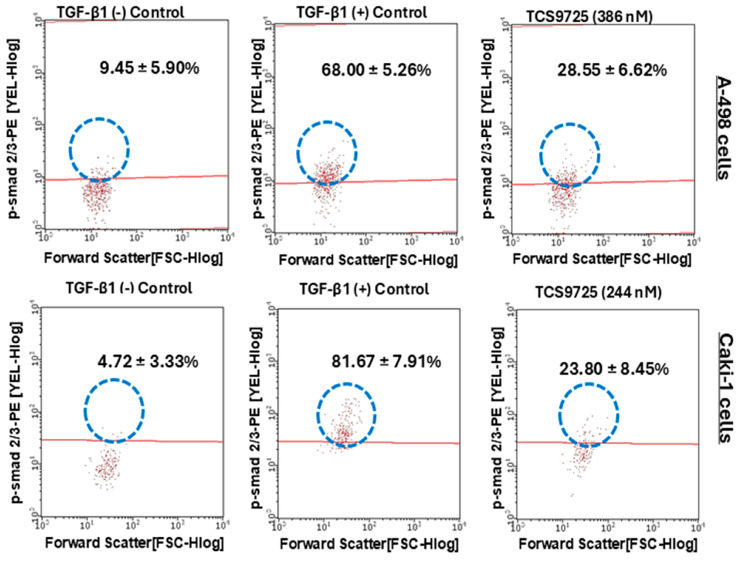
Effect of TCS9725 on p-smad 2/3 signaling. TGF-β (10 ng/mL) increased the smad2 (pS465/pS467)/smad3 (pS423/pS425)-positive population in A-498 and Caki-1 cells when compared to the TGF-β (−) control. TCS9725 downregulated TGF-β-stimulated phospho smad2/smad3 signaling in A-498 and Caki-1 cells. Representative graphs are depicted. Numerical values are mean ± SD from three individual experiments. Statistical significance at *p* < 0.05.

To demonstrate the mechanistic activity of TCS9725’s efficacy on the migration of RCC cells, we investigated TGF-β-stimulated smad2/3 signaling by flow cytometry. In the A-498 cells, 10 ng/mL TGF-β stimulated p-smad2/3-positive populations from 9.45 ± 5.90% to 68.00 ± 5.26% (Figure 11). Treatment with 386 nM TCS9725 reduced p-smad2/3-positive populations to 28.55 ± 6.62% in these cells (Figure 11). Caki-1 cells exhibited an 81.67 ± 7.91% p-samd2/3-positive population when stimulated with TGF-β, while the TGF-β (−) control exhibited a 4.72 ± 3.33% p-smad2/3-positive population (Figure 11). Treatment with 244 nM of TCS9725 reduced the p-smad2/3-positive populations to 23.80 ± 8.45% in these cells (Figure 11).

## 4. Discussion

The computational studies in this work focused on the identification of selective dual inhibition of TNKS1 and CDK8, aiming to attenuate the STAT1, β-Catenin, and TGFβ1-Smad cross-talk pathways. These signaling pathways are known to play crucial roles in renal cancer cell proliferation and metastasis, making TNKS1 and CDK8 promising therapeutic targets for cancer treatment. Through high-throughput virtual screening, TCS9725 was identified as a potent inhibitor of both TNKS1 and CDK8. Molecular docking and dynamics simulations demonstrated that TCS9725 forms stable interactions within the active sites of both proteins, engaging in hydrogen bonds, π-alkyl bonds, and hydrophobic interactions that favor ligand binding [27,28]. The RMSD and hydrogen bonding analysis over a 100 ns simulation confirmed the stability of these interactions, reinforcing TCS9725’s potential as a dual-target inhibitor. Furthermore, Gibbs’s free energy calculations using the MMPBSA method showed favorable binding affinities for TCS9725 with both TNKS1 and CDK8, supporting its efficacy in targeting these proteins. The ADMET properties predicted for TCS9725 suggest that it possesses favorable pharmacokinetic characteristics, such as good absorption, permeability, and metabolic stability, along with minimal toxicity concerns, making it a promising candidate for further development in cancer therapeutics [29].

By targeting both TNKS1 and CDK8, TCS9725 is expected to disrupt critical signaling pathways involved in renal cancer progression, including STAT1, β-Catenin, and TGFβ1-Smad signaling, which are known to drive cancer cell proliferation and metastasis.

In addition to the encouraging computational findings, TCS9725’s in vitro effectiveness was tested to confirm its promise as a TNKS1 and CDK8 dual inhibitor using enzymatic assays. Results revealed dose-dependent inhibition of both TNKS1 and CDK8 enzymes by TCS9725, demonstrating the compound’s capacity to effectively inhibit target activities at low doses. Small-molecule TNKS1 inhibitors were proven effective against tumor cell lines and mouse models. [30]. Despite advances in structural development, no TNKS1/2 inhibitor has yet entered clinical trials for any application. [30,31]. Concerns about intestinal toxicity and other on-target/signaling-pathway-specific side effects have impeded clinical tankyrase inhibitor development. Although current preclinical stage tankyrase-specific inhibitors, such as G007-LK, do not meet the chemical properties required for human testing, research to develop additional TNKS1/2 inhibitors for clinical use is ongoing. [32]. TNKS1 can coordinate the actions of numerous biological systems, such as proliferation, differentiation, and energy metabolism [7]. Deregulation of the Wnt/β-catenin signaling pathway by TNKS1 is linked to the development and progression of cancer. [33,34]. Treatment with XAV939, a tankyrase inhibitor, reduced the survival of cervical cancer cells while increasing radiosensitivity. [35]. Furthermore, the tankyrase-specific inhibitors JW74 and JW55 influence cell cycle progression and trigger apoptosis and differentiation in osteosarcoma and colon cancer cells [36]. In addition, mice xenografts and patient-derived sphere cultures of colorectal cancer (CRC) were treated with the tankyrase inhibitor NVP-TNKS656 in conjunction with AKT and PI3K inhibitors to downregulate β-catenin levels and induce apoptosis. [37]. Consistent with these observations, TCS9725 controlled the proliferation of RCC cells while downregulating the β-catenin levels and promoting apoptosis in these cells. While the aforementioned inhibitors have revealed concerns about toxicity [38], TCS9725 inhibited the viability of normal kidney cells at multifold concentrations that were required to control RCC cell proliferations. This suggested a higher therapeutic index and selectivity towards RCC cells by the compound.

On the other hand, several investigations have shown a strong relationship between malignancies and CDK8-mediated transcriptional regulation [39,40]. By controlling the kinase module’s catalytic activity, CDK8 enables it to attach to the mediator complex’s core structure and causes additional changes in functional activity [41]. According to recent research, CDK8 is essential for several signaling pathways, including the Wnt/β-catenin pathways that promote the spread of cancer cells [42]. As a result, researchers have focused a great deal of attention on CDK8, and several CDK8 inhibitors have been identified as promising cancer therapeutics. Focusing on the RCC, a combination of methylome and transcriptome analyses confirmed the mediators of the CDK8 complex to directly regulate the β-catenin-driven transcription of the Wnt/β-catenin signaling pathway in renal cancer samples [43]. Our observation was on par with this study, where TCS9725 inhibited CDK8 and downregulated β-catenin in RCC cells. However, we also checked other downstream events of CDK8 inhibition post-TCS9725 treatment. STAT1-S727 phosphorylation is frequently used to gauge CDK8/19 kinase activity since STAT1 marks a direct substrate of CDK8/19 [44]. CDK8-STAT1-mediated antitumor activity is also well documented [45,46,47]. Consistent with these studies, the phosphorylation of the CDK8 substrate STAT1 at the Ser727 site was inhibited by TCS9725 treatment in both cell lines tested.

Transforming Growth Factor-β (TGF-β) is a cytokine that has been widely investigated in tumor biology and is thought to play several roles in the growth of tumors [48]. TGF-β controls critical events of cancer metastasis. When RCC metastasizes, overexpression of TGF-β1 is substantially linked to a worse clinical prognosis [47]. We therefore checked the effect of TCS9725 on the migration of RCC cells under the influence of TGF-β1. TCS9725 inhibited the migration of RCC cells under the influence of TGF-β1. It is documented that CDK8 phosphorylates transcription factors to promote ligand-induced transcriptional activity [49]. The compound’s effects on the TGF-β/smad canonical pathway were investigated to determine whether the compound’s suppression of migration involves CDK8’s inhibition of transcriptional regulation. The smad family of signal transducers, including smad2 and smad3, undergo tail-phosphorylation at the C-terminal domain (e.g., Ser465/467) upon receptor activation by TGF-β1. These signal transducers subsequently form a complex with a comediator, smad4, which moves into the nucleus. Active RNA polymerase II S2/S5 follows the CDK8/mediator complex’s phosphorylation of smad3 in the nucleus to produce high-affinity binding sites for the coactivator Pin1 and other transcriptional partners, resulting in peak transcriptional activity that facilitates metastasis [50]. Our observation stands well with these observations, where TCS9725 reduced smad2/smad3 phosphorylation in RCC cells, favoring the anti-migratory effect by the compound mediated by the TGF-β/smad canonical pathway. Furthermore, the noncanonical pathway of TGF-β-CDK8 also contributes to the metastasis of some malignancies. A recent study showed that β-catenin-driven transcription requires CDK8 kinase activity [51]. Therefore, CDK8-β-catenin interplay in migration inhibition induced by TCS9725 cannot be sidelined.

Given the known roles of STAT1 and TGFβ in modulating immune responses [52,53], the inhibition of these pathways by TCS9725 may influence tumor–immune interactions, potentially altering immune cell activation, infiltration, or suppression within the tumor microenvironment [53]. While this study focused on tumor cell-intrinsic effects, future investigations employing co-culture systems or in vivo models with intact immune components will be essential to elucidate these effects. Such studies could provide important insights into the immunomodulatory potential of TCS9725 and its relevance in combination immunotherapy strategies.

While our observations affirm that dual inhibition of TNKS1 and CDK8 by TCS9725 involves STAT1, β-catenin, and TGF-β1-smad2/3 cross-talk in controlling renal cancer cells, we acknowledge that a key limitation of this study is the absence of in vivo validation to confirm the therapeutic potential of TCS9725 in a physiological context. Although our in vitro and computational findings provide a strong foundation, further preclinical investigations, including in vivo studies, are essential to evaluate pharmacokinetics, toxicity, and efficacy in a complex tumor microenvironment, and to elucidate details for the mechanism of action and develop this novel molecule as an effective chemotherapeutic drug against renal carcinoma.

## 5. Conclusions

To sum up, our results identified a novel TNKS1 and CDK8 dual inhibitor, TCS9725, possessing strong binding predictions towards targets with favorable drug characteristics. The compound effectively controlled the proliferation of RCC cells and downregulated STAT1 and β-catenin activities to favor apoptosis in these cells. TCS9725 inhibited TGF-β1-induced tumor migration and p-Smad2/3 activity in the RCC cells. From a computational perspective, the molecular docking and dynamics simulations revealed that TCS9725 binds stably within the active sites of both TNKS1 and CDK8, showing favorable interactions that were further supported by Gibbs’s free energy estimations. The ADMET predictions for TCS9725 suggest that the compound has good pharmacokinetic properties, including strong absorption, permeability, and metabolic stability, with minimal toxicity concerns. These results provide a strong foundation for the potential therapeutic application of TCS9725 as a dual inhibitor in the treatment of renal cancer. These preliminary findings open new ventures for preclinical and clinical investigations of TCS9725 and its analogs for further development. The computational analysis presented here also offers valuable insights into the design and optimization of future derivatives with enhanced efficacy and improved pharmacokinetic profiles.

## Data Availability

All data used in this manuscript are available from the author and can be provided upon reasonable request for noncommercial purposes.

## References

[1] Padala S.A., Barsouk A., Thandra K.C., Saginala K., Mohammed A., Vakiti A., Rawla P., Barsouk A. (2020). Epidemiology of Renal Cell Carcinoma. World J. Oncol..

[2] Banumathy G., Cairns P. (2010). Signaling pathways in renal cell carcinoma. Cancer Biol. Ther..

[3] Xu Q., Krause M., Samoylenko A., Vainio S. (2016). Wnt Signaling in Renal Cell Carcinoma. Cancers.

[4] Yip H.Y.K., Papa A. (2021). Signaling Pathways in Cancer: Therapeutic Targets, Combinatorial Treatments, and New Developments. Cells.

[5] Matuszczak M., Kiljańczyk A., Salagierski M. (2023). Surgical Approach in Metastatic Renal Cell Carcinoma: A Literature Review. Cancers.

[6] Martino-Echarri E., Brocardo M.G., Mills K.M., Henderson B.R. (2016). Tankyrase Inhibitors Stimulate the Ability of Tankyrases to Bind Axin and Drive Assembly of β-Catenin Degradation-Competent Axin Puncta. PLoS ONE.

[7] Haikarainen T., Krauss S., Lehtio L. (2014). Tankyrases: Structure, function and therapeutic implications in cancer. Curr. Pharm. Des..

[8] Fujita M., Demizu Y. (2024). Advances in the development of Wnt/β-catenin signaling inhibitors. RSC Med. Chem..

[9] Menck K., Heinrichs S., Baden C., Bleckmann A. (2021). The WNT/ROR Pathway in Cancer: From Signaling to Therapeutic Intervention. Cells.

[10] Friedson B., Willis S.D., Shcherbik N., Campbell A.N., Cooper K.F. (2025). The CDK8 kinase module: A novel player in the transcription of translation initiation and ribosomal genes. Mol. Biol. Cell.

[11] Bian J., Dannappel M., Wan C., Firestein R. (2020). Transcriptional Regulation of Wnt/β-Catenin Pathway in Colorectal Cancer. Cells.

[12] Chen M., Li J., Liang J., Thompson Z.S., Kathrein K., Broude E.V., Roninson I.B. (2019). Systemic Toxicity Reported for CDK8/19 Inhibitors CCT251921 and MSC2530818 Is Not Due to Target Inhibition. Cells.

[13] Arnett A., Moo K.G., Flynn K.J., Sundberg T.B., Johannessen L., Shamji A.F., Gray N.S., Decker T., Zheng Y., Gersuk V.H. (2021). The Cyclin-Dependent Kinase 8 (CDK8) Inhibitor DCA Promotes a Tolerogenic Chemical Immunophenotype in CD4(+) T Cells via a Novel CDK8-GATA3-FOXP3 Pathway. Mol. Cell. Biol..

[14] Yin X., He Z., Chen K., Ouyang K., Yang C., Li J., Tang H., Cai M. (2024). Unveiling the impact of CDK8 on tumor progression: Mechanisms and therapeutic strategies. Front. Pharmacol..

[15] Yesilkanal A.E., Johnson G.L., Ramos A.F., Rosner M.R. (2021). New strategies for targeting kinase networks in cancer. J. Biol. Chem..

[16] Yu F., Yu C., Li F., Zuo Y., Wang Y., Yao L., Wu C., Wang C., Ye L. (2021). Wnt/β-catenin signaling in cancers and targeted therapies. Signal Transduct. Target. Ther..

[17] Bayat Mokhtari R., Homayouni T.S., Baluch N., Morgatskaya E., Kumar S., Das B., Yeger H. (2017). Combination therapy in combating cancer. Oncotarget.

[18] Makhov P., Joshi S., Ghatalia P., Kutikov A., Uzzo R.G., Kolenko V.M. (2018). Resistance to Systemic Therapies in Clear Cell Renal Cell Carcinoma: Mechanisms and Management Strategies. Mol. Cancer Ther..

[19] Al Shahrani M., AboHassan M., Gahtani R., Alshahrani M.Y., Suliman M., Ahmad I., Saeed M. (2025). High-throughput screening and in vitro evaluation of CSB-0914; a novel small molecule NF-κB inhibitor attenuating inflammatory responses through NF-κB, Nrf2 and HO-1 cross-talk. J. Biomol. Struct. Dyn..

[20] Trott O., Olson A.J. (2010). AutoDock Vina: Improving the speed and accuracy of docking with a new scoring function, efficient optimization, and multithreading. J. Comput. Chem..

[21] Van Der Spoel D., Lindahl E., Hess B., Groenhof G., Mark A.E., Berendsen H.J. (2005). GROMACS: Fast, flexible, and free. J. Comput. Chem..

[22] Alghamdi M.A., Deshpande H. (2025). Dual Targeting of MEK1 and Akt Kinase Identified SBL-027 as a Promising Lead Candidate to Control Cell Proliferations in Gastric Cancer. Biotechnol. Appl. Biochem..

[23] Utami P.D., Setianingsih H., Sari D.R.T. (2024). In Silico Approach Triterpene Glycoside of H. atra Targeting Orotidine 5-Monophosphate Decarboxylase Protein (PfOMPDC) in P. falciparum Infection Mechanism. BioMed Res. Int..

[24] Swanson K., Walther P., Leitz J., Mukherjee S., Wu J.C., Shivnaraine R.V., Zou J. (2024). ADMET-AI: A machine learning ADMET platform for evaluation of large-scale chemical libraries. Bioinformatics.

[25] Abohassan M., Alshahrani M., Alshahrani M.Y., Rajagopalan P. (2022). Insilco and Invitro approaches identify novel dual PI3K/AKT pathway inhibitors to control acute myeloid leukemia cell proliferations. Med. Oncol..

[26] Al Shahrani M., Abohassan M., Alshahrani M., Gahtani R.M., Rajagopalan P. (2025). Identification of 8-(2-methyl phenyl)-9H-benzo[f]indeno [2,1-c]quinolin-9-one (C-5635020) as a novel and selective TGFβ RII kinase inhibitor for breast cancer therapy. Biochem. Biophys. Res. Commun..

[27] Halder S.K., Sultana I., Shuvo M.N., Shil A., Himel M.K., Hasan M.A., Shawan M. (2023). In Silico Identification and Analysis of Potentially Bioactive Antiviral Phytochemicals against SARS-CoV-2: A Molecular Docking and Dynamics Simulation Approach. BioMed Res. Int..

[28] Santos L.H.S., Ferreira R.S., Caffarena E.R. (2019). Integrating Molecular Docking and Molecular Dynamics Simulations. Methods Mol. Biol..

[29] Vora L.K., Gholap A.D., Jetha K., Thakur R.R.S., Solanki H.K., Chavda V.P. (2023). Artificial Intelligence in Pharmaceutical Technology and Drug Delivery Design. Pharmaceutics.

[30] Mygland L., Brinch S.A., Strand M.F., Olsen P.A., Aizenshtadt A., Lund K., Solberg N.T., Lycke M., Thorvaldsen T.E., Espada S. (2021). Identification of response signatures for tankyrase inhibitor treatment in tumor cell lines. iScience.

[31] Zhong L., Ding Y., Bandyopadhyay G., Waaler J., Börgeson E., Smith S., Zhang M., Phillips S.A., Mahooti S., Mahata S.K. (2016). The PARsylation activity of tankyrase in adipose tissue modulates systemic glucose metabolism in mice. Diabetologia.

[32] Voronkov A., Holsworth D.D., Waaler J., Wilson S.R., Ekblad B., Perdreau-Dahl H., Dinh H., Drewes G., Hopf C., Morth J.P. (2013). Structural basis and SAR for G007-LK, a lead stage 1,2,4-triazole based specific tankyrase 1/2 inhibitor. J. Med. Chem..

[33] Zhang M., Weng W., Zhang Q., Wu Y., Ni S., Tan C., Xu M., Sun H., Liu C., Wei P. (2018). The lncRNA NEAT1 activates Wnt/β-catenin signaling and promotes colorectal cancer progression via interacting with DDX5. J. Hematol. Oncol..

[34] Cao M.Q., You A.B., Zhu X.D., Zhang W., Zhang Y.Y., Zhang S.Z., Zhang K.W., Cai H., Shi W.K., Li X.L. (2018). miR-182-5p promotes hepatocellular carcinoma progression by repressing FOXO3a. J. Hematol. Oncol..

[35] Zhang J., Si J., Gan L., Guo M., Yan J., Chen Y., Wang F., Xie Y., Wang Y., Zhang H. (2020). Inhibition of Wnt signalling pathway by XAV939 enhances radiosensitivity in human cervical cancer HeLa cells. Artif. Cells Nanomed. Biotechnol..

[36] Stratford E.W., Daffinrud J., Munthe E., Castro R., Waaler J., Krauss S., Myklebost O. (2014). The tankyrase-specific inhibitor JW74 affects cell cycle progression and induces apoptosis and differentiation in osteosarcoma cell lines. Cancer Med..

[37] Arqués O., Chicote I., Puig I., Tenbaum S.P., Argilés G., Dienstmann R., Fernández N., Caratù G., Matito J., Silberschmidt D. (2016). Tankyrase Inhibition Blocks Wnt/β-Catenin Pathway and Reverts Resistance to PI3K and AKT Inhibitors in the Treatment of Colorectal Cancer. Clin. Cancer Res..

[38] Thorne C.A., Hanson A.J., Schneider J., Tahinci E., Orton D., Cselenyi C.S., Jernigan K.K., Meyers K.C., Hang B.I., Waterson A.G. (2010). Small-molecule inhibition of Wnt signaling through activation of casein kinase 1α. Nat. Chem. Biol..

[39] Ho T.-Y., Sung T.-Y., Pan S.-L., Huang W.-J., Hsu K.-C., Hsu J.-Y., Lin T.E., Hsu C.-M., Yang C.-R. (2023). The study of a novel CDK8 inhibitor E966-0530–45418 that inhibits prostate cancer metastasis in vitro and in vivo. Biomed. Pharmacother..

[40] Brägelmann J., Klümper N., Offermann A., von Mässenhausen A., Böhm D., Deng M., Queisser A., Sanders C., Syring I., Merseburger A.S. (2017). Pan-Cancer Analysis of the Mediator Complex Transcriptome Identifies CDK19 and CDK8 as Therapeutic Targets in Advanced Prostate Cancer. Clin. Cancer Res..

[41] Allen B.L., Taatjes D.J. (2015). The Mediator complex: A central integrator of transcription. Nat. Rev. Mol. Cell Biol..

[42] Calon A., Espinet E., Palomo-Ponce S., Tauriello D.V., Iglesias M., Céspedes M.V., Sevillano M., Nadal C., Jung P., Zhang X.H. (2012). Dependency of Colorectal Cancer on a TGF-β-Driven Program in Stromal Cells for Metastasis Initiation. Cancer Cell.

[43] Arai E., Sakamoto H., Ichikawa H., Totsuka H., Chiku S., Gotoh M., Mori T., Nakatani T., Ohnami S., Nakagawa T. (2014). Multilayer-omics analysis of renal cell carcinoma, including the whole exome, methylome and transcriptome. Int. J. Cancer.

[44] Bancerek J., Poss Z.C., Steinparzer I., Sedlyarov V., Pfaffenwimmer T., Mikulic I., Dölken L., Strobl B., Müller M., Taatjes D.J. (2013). CDK8 Kinase Phosphorylates Transcription Factor STAT1 to Selectively Regulate the Interferon Response. Immunity.

[45] Lee J.C., Liu S., Wang Y., Liang Y., Jablons D.M. (2022). MK256 is a novel CDK8 inhibitor with potent antitumor activity in AML through downregulation of the STAT pathway. Oncotarget.

[46] Spear J.M., Lu Z., Russu W.A. (2020). Pharmacological Inhibition of CDK8 in Triple-Negative Breast Cancer Cell Line MDA-MB-468 Increases E2F1 Protein, Induces Phosphorylation of STAT3 and Apoptosis. Molecules.

[47] Solum E., Hansen T.V., Aesoy R., Herfindal L. (2020). New CDK8 inhibitors as potential anti-leukemic agents—Design, synthesis and biological evaluation. Bioorganic Med. Chem..

[48] Boström A.-K., Lindgren D., Johansson M.E., Axelson H. (2013). Effects of TGF-β signaling in clear cell renal cell carcinoma cells. Biochem. Biophys. Res. Commun..

[49] Menzl I., Witalisz-Siepracka A., Sexl V. (2019). CDK8-Novel Therapeutic Opportunities. Pharmaceuticals.

[50] Alarcón C., Zaromytidou A.-I., Xi Q., Gao S., Yu J., Fujisawa S., Barlas A., Miller A.N., Manova-Todorova K., Macias M.J. (2009). Nuclear CDKs Drive Smad Transcriptional Activation and Turnover in BMP and TGF-β Pathways. Cell.

[51] Firestein R., Bass A.J., Kim S.Y., Dunn I.F., Silver S.J., Guney I., Freed E., Ligon A.H., Vena N., Ogino S. (2008). CDK8 is a colorectal cancer oncogene that regulates β-catenin activity. Nature.

[52] Tolomeo M., Cavalli A., Cascio A. (2022). STAT1 and Its Crucial Role in the Control of Viral Infections. Int. J. Mol. Sci..

[53] Sanjabi S., Oh S.A., Li M.O. (2017). Regulation of the Immune Response by TGF-β: From Conception to Autoimmunity and Infection. Cold Spring Harb. Perspect. Biol..

